# An analysis of intersectional disparities in alcohol consumption in the US

**DOI:** 10.1016/j.socscimed.2024.117514

**Published:** 2024-11-15

**Authors:** Sophie Bright, Charlotte Buckley, Daniel Holman, George Leckie, Andrew Bell, Nina Mulia, Carolin Kilian, Robin Purshouse

**Affiliations:** a Sheffield Centre for Health and Related Research (ScHARR), Faculty of Medicine, Dentistry & Health, University of Sheffield, Sheffield, UK; b Department of Automatic Control and Systems Engineering, University of Sheffield, UK; c Department of Sociological Studies, University of Sheffield, Sheffield, UK; d Centre for Multilevel Modelling and School of Education, University of Bristol, UK; e Sheffield Methods Institute, University of Sheffield, Sheffield, UK; f Alcohol Research Group, Public Health Institute, Emeryville, CA, USA; g Institute for Mental Health Policy Research, Centre for Addiction and Mental Health, Toronto, Ontario, Canada; h Center for Interdisciplinary Addiction Research (ZIS), Department of Psychiatry and Psychotherapy, University Medical Center Hamburg-Eppendorf (UKE), Hamburg, Germany; i Department of Psychology, Institute of Population Health, University of Liverpool

**Keywords:** Alcohol, Health inequities, Intersectionality, MAIHDA, Multilevel analysis of individual heterogeneity and discriminatory accuracy, Multilevel analysis, Substance misuse, United States

## Abstract

Alcohol is one of the leading causes of preventable deaths in the United States (US). Prior research has demonstrated that alcohol consumption and related mortality are socially patterned; however, no study has investigated intersectional disparities in alcohol consumption, i.e., attending to how social positions overlap and interact. In this study, we used an innovative intersectional approach (Multilevel Analysis of Individual Heterogeneity and Discriminatory Accuracy, MAIHDA) and data from a large nationally representative survey (the National Health Interview Survey, 2000–2018) to quantify inter-categorical disparities in alcohol consumption in the US (proportion of current drinkers, and average consumption amongst drinkers), along dimensions of sex, race and ethnicity, age, and level of education. Our analysis revealed significant intersectional disparities in both the prevalence of drinking and the average consumption by drinkers. Young, highly educated White men were the most likely to be current drinkers and consumed the highest amounts of alcohol on average, whilst racially and ethnically minoritized women with lower education were the least likely to drink and had the lowest levels of alcohol consumption, across all age categories. Notably, we found significant interaction effects for many intersectional strata, with much higher consumption estimated for some groups than traditional additive approaches would suggest. By identifying specific understudied groups with high consumption, such as young American Indian or Alaska Native (AI/AN) men, adult Black men with low education, and older White women with high education, this analysis has important implications for future research, policy, and praxis. This is the first known application of MAIHDA to account for a skewed outcome, highlighting and addressing critical methodological considerations.

## 1. Introduction

Alcohol is a leading cause of preventable deaths in the United States (US) (CDC, 2022). Alcohol consumption is socially patterned in the US, differing by gender, race and ethnicity, age, and socio-economic status (SES). For example, in unitary terms, men drink more than women (White, 2020), individuals generally drink more heavily when they are younger than when they are older (Karlamangla et al., 2006), and those with high education tend to drink more than those with low education (Lui et al., 2018). Non-Hispanic White people are the most likely racial/ethnic group to be current drinkers, whilst non-Hispanic American Indian/Alaska Native (AI/AN) people are most likely to binge drink and drink heavily (NIAAA, 2024). Whilst these broad trends provide useful understanding of differences in alcohol consumption, there is substantial complexity and nuance beyond these single-axes differences. Further, whilst individuals holding more privileged social positions, such as higher levels of education and White race, are more likely to drink alcohol, disparities in alcohol-related harm manifest in complex ways. Individuals with lower education and racialized or minoritized groups experience disproportionately higher alcohol-attributable harms despite lower or equivalent alcohol consumption (Probst et al., 2021; Zapolski et al., 2014). A better understanding of consumption from an intersectional perspective, combined with future intersectional analyses on harm, could help identify groups with the greatest disparity between consumption and harm, thereby guiding targeted interventions to support both high-exposure and high-vulnerability groups.

A limited number of existing studies have provided insights into how social positions overlap and interact in relation to alcohol use in the US (Eisenberg et al., 2022; Glass et al., 2017; Greene et al., 2020). For example, Glass et al., explored multiplicative interactions between poverty, sex, and race and ethnicity, finding that the effect of poverty on past-year incidence of heavy episodic drinking (HED) was stronger among Black men and women in comparison to men and women of other racial and ethnic groups (Glass et al., 2017). However, no known analysis has comprehensively mapped differences in alcohol consumption at the intersection of sex, race and ethnicity, age, and education simultaneously.

Intersectionality is a critical analytic framework grounded in Black feminist history and activism (Combahee River Collective, 1977; Crenshaw, 1989; Hill Collins, 1991), increasingly recognised as important for public health (Bowleg, 2012). It emphasises that social positions – such as race, gender, and class – are not held in isolation, but instead overlap and interact. Intersectionality focuses on how power, privilege, oppression, and discrimination (such as racism, sexism, and classism), intersect to create complex social inequalities, and highlights how wider social structures, such as the political, educational, and legal systems, contribute to these inequalities. These factors are known to have a bearing on alcohol consumption, for example, through differential exposure to chronic and acute stressors, alcohol advertising and outlet density, and access to resources for health and well-being. A critical intersectional perspective is therefore useful for investigating disparities in alcohol consumption.

Multilevel Analysis of Individual Heterogeneity and Discriminatory Accuracy (MAIHDA) (Evans et al., 2018) – a form of multilevel regression modelling used for inter-categorical intersectional analyses – is recognised as an important method for investigating health disparities in social epidemiology research (Merlo, 2018). MAIHDA clusters individuals within intersectional groups (or ‘strata’), based on their sociodemographic characteristics (e.g., ‘White, female, low education’), reflecting that individuals within one stratum share a similar social environment e.g., sociocultural norms, exposure to discrimination, and access to resources. The social positions most commonly considered within quantitative intersectional analysis are sex/gender, race/ethnicity, socioeconomic status, sexual orientation, and age/generation, all of which have clear ties to social power (Bauer et al., 2021). MAIHDA has already been applied to several health outcomes, such as body mass index (Evans et al., 2018), depression (Evans, 2019), and birthweight (Nieves et al., 2023), with studies often considering at least three of the abovementioned social positions, sometimes in combination with others. However, despite literature indicating the unequal distribution of alcohol use, to the best of our knowledge, MAIHDA has not been applied to the study of alcohol consumption.

### 1.1. Research question and aims

In this study, we aim to address the question: “To what extent are there differences in alcohol consumption within and between intersectional social strata?”

Specifically, our aims are:

1. To comprehensively map out alcohol consumption levels across multiple intersectional strata, increasing understanding of alcohol consumption among often-invisible populations, and identifying who drinks the least and the most.
2. To identify groups for whom there are significant interaction effects, i.e., who drinks more/less than might be expected given the additive combination of their social positions.

It is important to note that this analysis seeks to understand how different social positions combine to influence alcohol consumption outcomes, without necessarily attributing causality to specific factors.

## 2. Methods

We used a descriptive, inter-categorical intersectional approach to analyse disparities in alcohol consumption between social groups (McCall, 2005). We used MAIHDA models to examine the effect of intersecting social positions on alcohol consumption, considering the influence of both additive (layering) and multiplicative (interaction) effects. We explored the intersections of two sex, six race and ethnicity, three age, and three educational attainment categories, resulting in 108 intersectional strata (2 × 6 × 3 × 3). We first predicted the proportion of current drinkers within each stratum, and then predicted the average alcohol consumption (grams of pure alcohol per day) for current drinkers.

There are several quantitative methods available for conducting intersectional analysis, including MAIHDA, cross-classification, regression with interactions, and decision tree methods. Each of these approaches has its strengths and limitations; however, no single method inherently ensures that research is intersectional. Intersectionality requires not only the use of appropriate methods but also contextualizing results within broader systems of power and oppression (Guan et al., 2021). A key strength of MAIHDA is that it allows for numerous interactions between social categories (Hancock, 2007) and produces more accurate estimates even in the presence of small samples (Mahendran et al., 2022a; Van Dusen et al., 2024). Specifically, MAIHDA shrinks predicted values for each stratum towards the additive predictions based on their sample size, addressing concerns about multiple testing, and reducing the risk of overinterpreting findings (Bell et al., 2019). In a recent study evaluating seven quantitative methods for conducting descriptive intersectional analysis with binary health outcomes, including classification and regression trees (CART) and random forests (Mahendran et al., 2022b), found that MAIHDA produced the most accurate intersection-specific estimates, particularly in studies with small sample sizes. MAIHDA also requires fewer regression coefficients than the equivalent single-level models with interaction terms, making MAIHDA models more parsimonious and computationally efficient. Finally, MAIHDA allows for partitioning outcome variance within and between strata, providing a deeper understanding of population variability and disparities and offering a measure of the discriminatory accuracy of the strata categorizations used. Therefore, MAIHDA supports a nuanced understanding of health issues and reduces the risk of drawing incorrect conclusions about health equity (Bauer and Scheim, 2019).

### 2.1. Data source and data processing

We used publicly available data from the US National Health Interview Survey (NHIS) – an annual repeated cross-sectional household interview of the civilian non-institutionalized population (NHIS, 2022) – for the years 2000–2018, accessed via the IPUMS USA database (Ruggles et al., 2023). Since no alcohol data were available for 2019 (due to a change in survey design) and drinking patterns appear to have changed during the COVID-19 pandemic (Leventhal et al., 2022), we did not include data from 2019 onward. Wording of the survey questions for race, ethnicity and sex remained the same across the sample period.

The NHIS population of ‘sample adults’ surveyed between 2000 and 2018 was 572,339 (one ‘sample adult’ aged 18 years or over is randomly selected from each household, and asked questions including about alcohol consumption). We excluded individuals under 21 years old (the legal drinking age in the US, n = 21,360), those categorized as ‘other’ race and ethnicity (n = 796), and those with missing education data (n = 4,245) or current drinking status (n = 9,969). For the binary analysis of drinking status, this provided an analytic sample of n = 526,557.

**Fig. 1.**
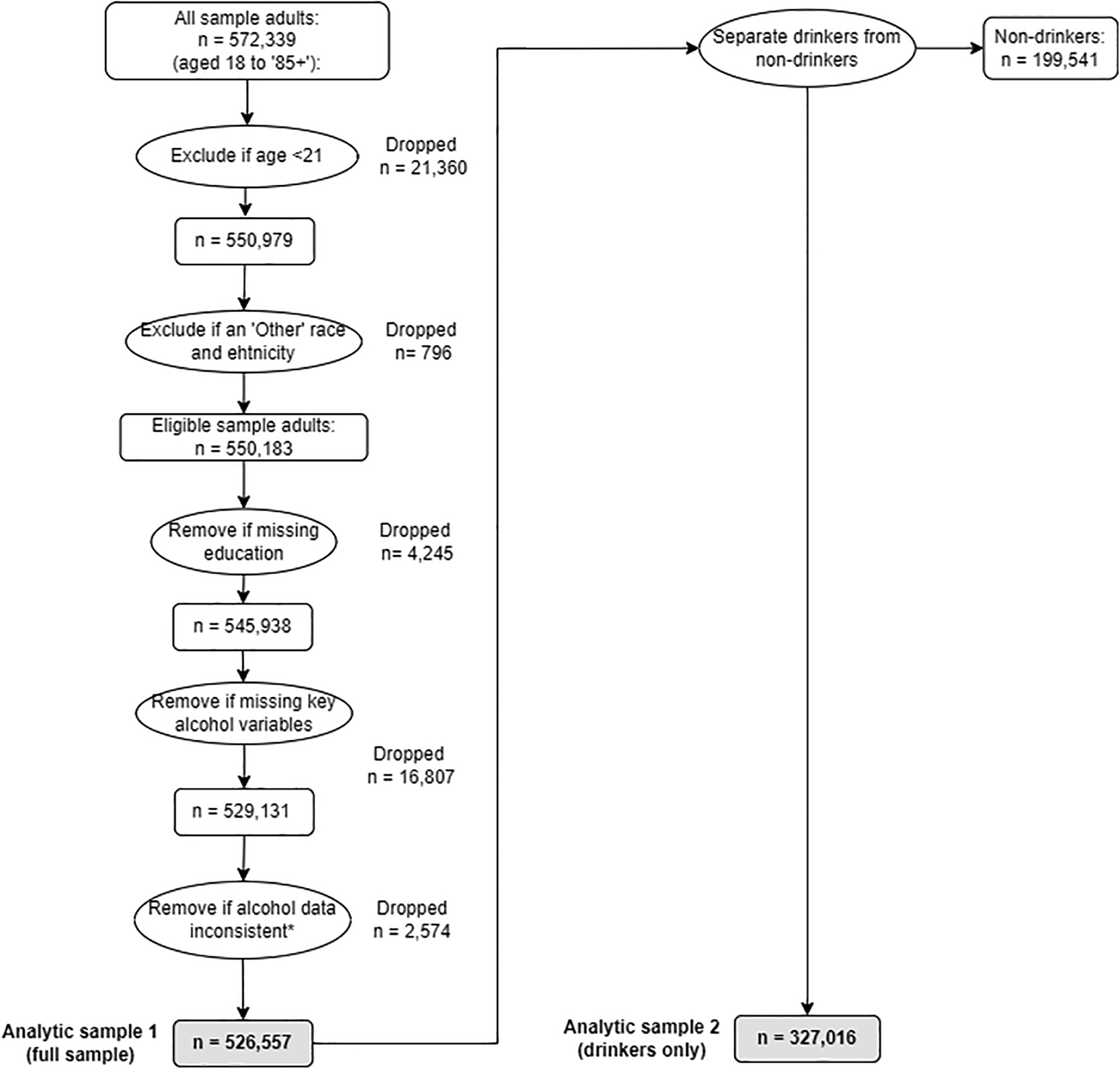
Process of removing missing data and sub-setting sample.

For the analysis of average consumption among current drinkers, we further excluded individuals who were non-drinkers (n = 199,541) and those with missing (n = 6,838) or inconsistent (n = 2,574) drinking quantity/frequency data, leaving an analytical sample of n = 327,016 (see Fig. 1). Individuals with missing data were excluded from the analysis because they comprised a small proportion of the sample (n = 23,626, 4.3% of eligible sample adults) and there were no substantial differences in missing data among demographic groups, based on visual inspection of boxplots.

### 2.2. Intersectional groups

MAIHDA analyses allow for the simultaneous consideration of numerous strata, and it is not uncommon for studies to include more than 100 strata e.g., Nieves et al. (2023). We considered 108 strata, defined by sex, race and ethnicity, socioeconomic status, and age. While many social positions could have been included (e.g., nativity, language preference), the variables selected were chosen due to their well-documented association with disparities in alcohol consumption in isolation, their relationship with social power, and are their role as key axes of social inequality.

#### 2.2.1. Sex

The NHIS variable ‘sex’ indicates whether a person is male or female, based on self-report or the interviewer’s best guess. The survey does not distinguish between sex and gender identity or allows for non-binary identities. We interpret responses as most indicative of sex but acknowledge the substantial limitations of this variable and its interpretation.

#### 2.2.2. Race and ethnicity

We generated a variable of ‘Race and ethnicity’ by combining responses from two self-reported closed-response questions (Race and Hispanic origin). Respondents who selected more than one racial category were coded as “multiple race” by the interviewer. We created six ‘race and ethnicity’ categories: Non-Hispanic (NH) White; NH Black; NH Asian; NH American Indian/Alaska Native; NH multiracial; and Hispanic (referred without the NH prefix hereafter). Whilst we recognise the substantial heterogeneity within these subgroups, particularly due to the lack of racial disaggregation within ‘Hispanic’, these six groupings were chosen based on the resulting subgroup sample sizes and the interpretability and meaningfulness of results. Overall, this method resulted in the exclusion of only two relatively small sub-groups: ‘non-Hispanic other race’ (n = 115) and ‘Non-Hispanic race group not releasable’ (n = 681).

#### 2.2.3. Educational attainment

Education was a categorical variable with three levels: high school or less, some college, and 4+ years of college. These cut-offs reflect key educational milestones in the US.

#### 2.2.4. Age

Age was categorized into three groups based on suggested standardised age groupings (Diaz et al., 2021): 21–24 (‘young adults’), 25–59 (‘adults’) and 60+ (‘older adults’). While further disaggregation might have provided additional insights, these capture the most significant changes in drinking over the life course, while maintaining viable strata sizes. All adults over the age of 85 years were recorded as ‘85+’ within the NHIS, so the exact upper age of the sample is unknown.

### 2.3. Outcomes

We considered two outcomes, current drinking status and the average alcohol consumption of drinkers. Current drinking status was operationalised as a binary variable: ‘current drinkers’ (drank at least once in the last 12 months) and ‘non-drinkers’ (no alcohol in the last 12 months, including former drinkers and lifetime abstainers).

Average alcohol consumption was measured in grams of pure alcohol per day (GPD), derived from participants’ self-reported alcohol intake and generated using a combination of the basic quantity/frequency and expanded quantity/frequency approaches, assuming a standard drink contains 14 g of pure alcohol (see Appendix B). A cap of 200 g (~14 standard drinks) was applied to daily alcohol intake estimates to adjust unrealistic outliers (n = 188). As is typical, average consumption was positively skewed even after excluding non-drinkers, due to a large number of low-use drinkers. As such, we log-transformed the outcome prior to modelling (see 2.4.1 for further detail).

### 2.4. Analysis

#### 2.4.1. MAIHDA models

We used random-intercept intersectional MAIHDA models (Evans et al., 2024a,b) to estimate each outcome for 108 intersectional strata. Null models nested individuals within intersections, followed by main effects models incorporating fixed effects for demographic categories (sex, race and ethnicity, age, and level of education). Fixed effects dummy variables for each survey year were included to control for temporal changes in consumption.

It is important to note that this analysis seeks to understand how different social positions combine to influence alcohol consumption outcomes, without necessarily attributing causality to specific factors. Therefore, as is typical in intersectional MAIHDA, no additional covariates were included in the models, beyond the intersecting social categories of interest (Evans et al., 2024b). Including potential confounders (such as income or religious affiliation) might obscure the inequalities that MAIHDA is designed to identify, as many of these “confounders” are themselves influenced by the same structural factors tied to the social positions being studied. It is therefore recognised that any differences between strata may in part reflect differences in other unmeasured variables (such as nativity, English-speaking status, or sexual orientation).

We estimated the proportion of current drinkers in each stratum using logistic MAIHDA models. To estimate the average alcohol consumption of current drinkers, we applied linear MAIHDA models, log-transforming the average alcohol consumption prior to modelling to address its positive skew. We then used a three-step approach to back-transform the model predictions onto the original outcome scale. For details, see Appendix B.

We employed Bayesian Markov Chain Monte Carlo (MCMC) estimation procedures with ‘diffuse’ or ‘flat’ prior belief distributions for all models. MCMC methods were chosen over maximum likelihood estimation for their simplification of calculating 95% credible intervals (CIs) for complex non-linear functions of model parameters. Because MCMC methods do not generally accommodate survey weights, unweighted estimates are presented. See Appendix B for further statistical details.

#### 2.4.2. Estimation of outcomes at each intersection

We used the main effects model to generate overall predictions for each outcome for each stratum, incorporating both main and interactive effects, and to predict outcomes based on the main effects only. Interaction effects were isolated by subtracting main-effect predictions from total predictions, to allow these effects to be shown on the original scale of the dependent variable. Positive interaction effects indicate higher predictions than would be expected based on additive effects alone, whereas negative effects imply lower predictions. To predict the average alcohol consumption, we modelled the log-transformed variable and then back transformed the outputs (see Appendix B for further information on the statistical methods).

#### 2.4.3. Outcome heterogeneity within and between strata

For each model, we calculated the variance partition coefficient (VPC) to quantify variance at the intersectional group level and differentiate between additive and multiplicative effects, where a VPC greater than 0% in the main effects model indicates the presence of multiplicative effects. To calculate VPC in the logistic regression models, we followed the widely used latent response approach, setting $\sigma_{e}^{2}$ as equal to the variance of the standard logistic distribution ($\frac{\pi^{3}}{3}\approx 3.29$) (Evans et al., 2024b). We also calculated the percentage of proportional change in variance (PCV) to quantify the proportion of outcome variation between strata that is attributable to main effects (Rodriguez-Lopez et al., 2023) (see Appendix B for equations).

#### 2.4.4. Correlation between outcomes

We produced a scatter plot, with points for each strata and a linear regression line, to visualize the correlation between two predicted outcomes. This allowed us to identify whether groups who had more current drinkers also had higher average consumption among drinkers, and any variations from the trend. Outliers (classified as groups with residuals greater than twice the standard deviation) were identified by comparing observed and predicted values. We also calculated the correlation coefficient.

#### 2.4.5. Software

Analyses were conducted in R 4.2.2 using the R2MLwiN package (version ‘0.8.8’) (Zhang et al., 2016). Code to replicate all analyses is publicly available on GitHub (at https://github.com/sophiefeldmanbright/MAIHDA_alcohol).

## 3. Results

### 3.1. Analytic sample

Table 1 presents the characteristics of the sample in unitary terms. The majority of participants were aged 25–59, White, women with high school education or less. Strata size ranged from n = 2 to n = 32,671 (see Supplementary Table 1). Only three strata (2.7%) had an n < 20, all of which were American Indian/Alaska Native groups. As noted earlier, the estimates for these smaller groups are subject to greater shrinkage, which reduces the risk of spurious results.

### 3.2. Model parameters

Table 2 displays the regression coefficients and within- and between-stratum variance estimates for all models (tables including the coefficients for survey years are provided in Appendix A). The intercepts in the null models reflect the precision-weighted grand means of the stratum means. The fixed effect values in the main effects models reflect the additive patterns predicted by the social positions. Additive demographic variables were observed to act in the same direction in relation to both outcomes, with female sex, older age, non-White race and ethnicity, and lower levels of education being associated with lower estimates of drinking and consumption.

In the models of drinking status, coefficients are estimated on the log-odds scale. Exponentiating the coefficients gives odds ratio interpretation. For example, in the main effects model, the female coefficient is −0.55 giving an odds ratio of 0.57 = exp(−0.55). Thus, women have 0.57 times the odds of being current drinkers compared to men. In the log-linear models of average alcohol consumption, coefficients are estimated on the log scale. Exponentiating the coefficients gives percentage change interpretation. For example, in the main effects model, the female coefficient is −0.98 giving an exponentiated coefficient of 0.38 = exp(−0.98). Thus, women are predicted to consume 62% less alcohol than men (0.62 = 1–0.38).

VPCs reflect the total variance at the stratum level (16.8% in the drinking status model and 12.0% in the average consumption model), with the remaining variation (83.2% and 88% respectively) reflecting within-stratum heterogeneity. PCVs indicate the proportion of this stratum-level variance accounted for by additive main effects on the estimation metric scales (91.6% and 88.7%, respectively). The remaining unexplained variance, indicated by the VPC in the main effects models, reflects the variance attributed to the interaction effects (1.4% in both models).

### 3.3. Drinking status

**Fig. 2.**
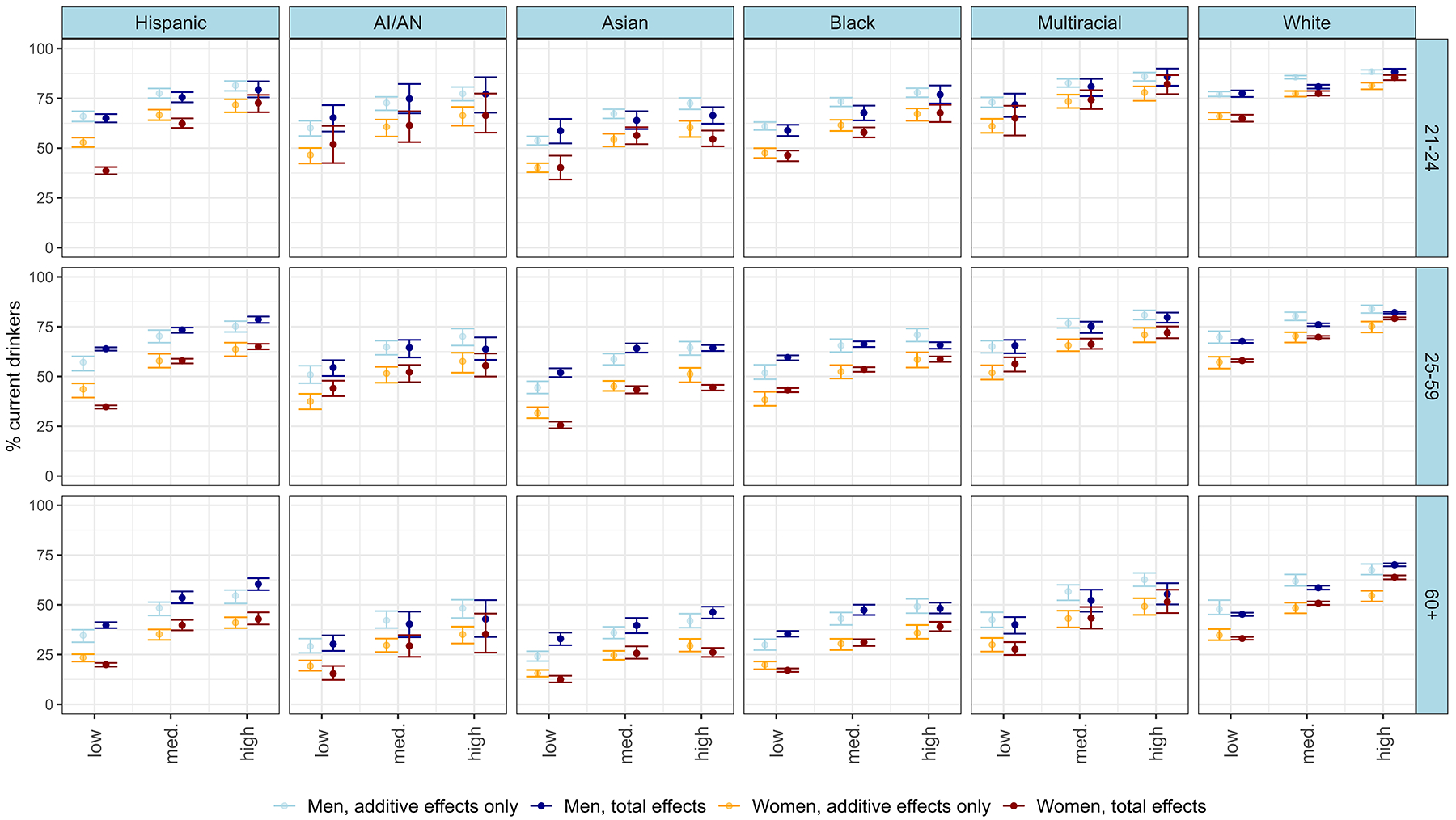
Plot of the predicted proportions of current drinkers in each intersectional stratum, with error bars indicating 95% credible intervals (comparing predictions based on total effects versus additive effects only).

In Fig. 2, we present the predicted proportions of current drinkers within different intersectional groups, along with 95% credible intervals. The figure contrasts the overall estimates with those derived solely from additive effects. Specifically, we represent estimates based solely on additive effects in light blue (for men) and orange (for women), while the overall ‘best’ estimates, incorporating both additive and multiplicative effects, are depicted in dark blue (for men) and red (for women). These results are also demonstrated in Table 3, which shows the estimated proportion of current drinkers (and 95% CI) for the five strata with i) the highest estimates, ii) the lowest estimates, iii) the largest positive interactions (i.e., higher estimates than would be expected based on additive effects only), and iv) the largest negative interactions (i.e., lower estimates than would be expected based on additive effects only). Estimates for all 108 strata are provided in Supplementary Table 1.

Comparing the total estimates (i.e., those accounting for both additive and interaction effects), we found significant variation in the proportion of current drinkers across strata, ranging from 12.5% (among older Asian women with low education) to 88.3% (among young White men with high education). In general, young, highly educated, White, and mixed-race men were the most likely to be current drinkers, while older women with low education, who belonged to minoritized race and ethnicity groups (Asian, AI/AN, Black, or Hispanic), were the least likely. Within a group, the most significant heterogeneity was observed among young women. For instance, only 38.6% of young Hispanic women with low education were current drinkers, compared to 85.4% of young White women with high education.

Several consistent trends also emerged across all intersectional groups, in line with the main effects. Firstly, men consistently exhibit higher proportions of current drinkers compared to women across all age, race, ethnicity, and education categories. Secondly, there is a clear educational gradient, with higher education levels correlating with higher likelihoods of being a current drinker, consistently observed across various demographic groups. Thirdly, young adults are consistently more likely to be current drinkers compared to older adults.

However, there are also important nuances within these findings, with the strength of each trend varying depending on other characteristics within a given stratum. For instance, concerning sex differences, young Hispanic men with low education are almost twice as likely to be current drinkers as young Hispanic women (64.9% vs. 38.6%, respectively), whereas the disparity diminishes among young White individuals with high education levels. Similarly, the educational gradient is notably stronger among young adult and adult Hispanic women compared to other groups. Additionally, the age gradient is most pronounced among AI/AN women with low education, where the proportion of current drinkers drops by 36.5% between the youngest and oldest categories, while being least noticeable among White men with high education, with a drop of 22.2%.

Significant interaction effects (indicated by 95% credible intervals not including zero) were found for 37 out of the 108 strata. In some instances, the interaction effects acted in the same direction as main effects. For example, White young women with high education were estimated to have a relatively high proportion of current drinkers based on additive effects (81.5%), and also had positive interaction effects (+3.9% = 85.4%). Similarly, for several groups of older, minoritized race and ethnicity, women, with low education, already low additive estimates were further lowered by negative interaction effects. However, we also found that some of the strongest interaction effects were for groups at neither extreme of the distribution. For example, young Hispanic women with low education, had the largest *negative* interaction effects (−14.3%), whilst older White women with low education exhibited the largest *positive* interaction effects (+9.2%). In other words, traditional, non-intersectional analyses, would *overestimate* the proportion of drinkers among young Latinas with low education, and *underestimate* it among older White women with low education. In one instance (for Black, adult, women with low education) the interaction effects acted in the opposite direction to the main effects. That is, whilst each of their social positions individually would predict lower consumption than the reference groups, the interaction effects for this group were positive, i.e., resulted in *less low* predictions than the additive effects would imply.

### 3.4. Average alcohol consumption: grams of pure alcohol per day

**Fig. 3.**
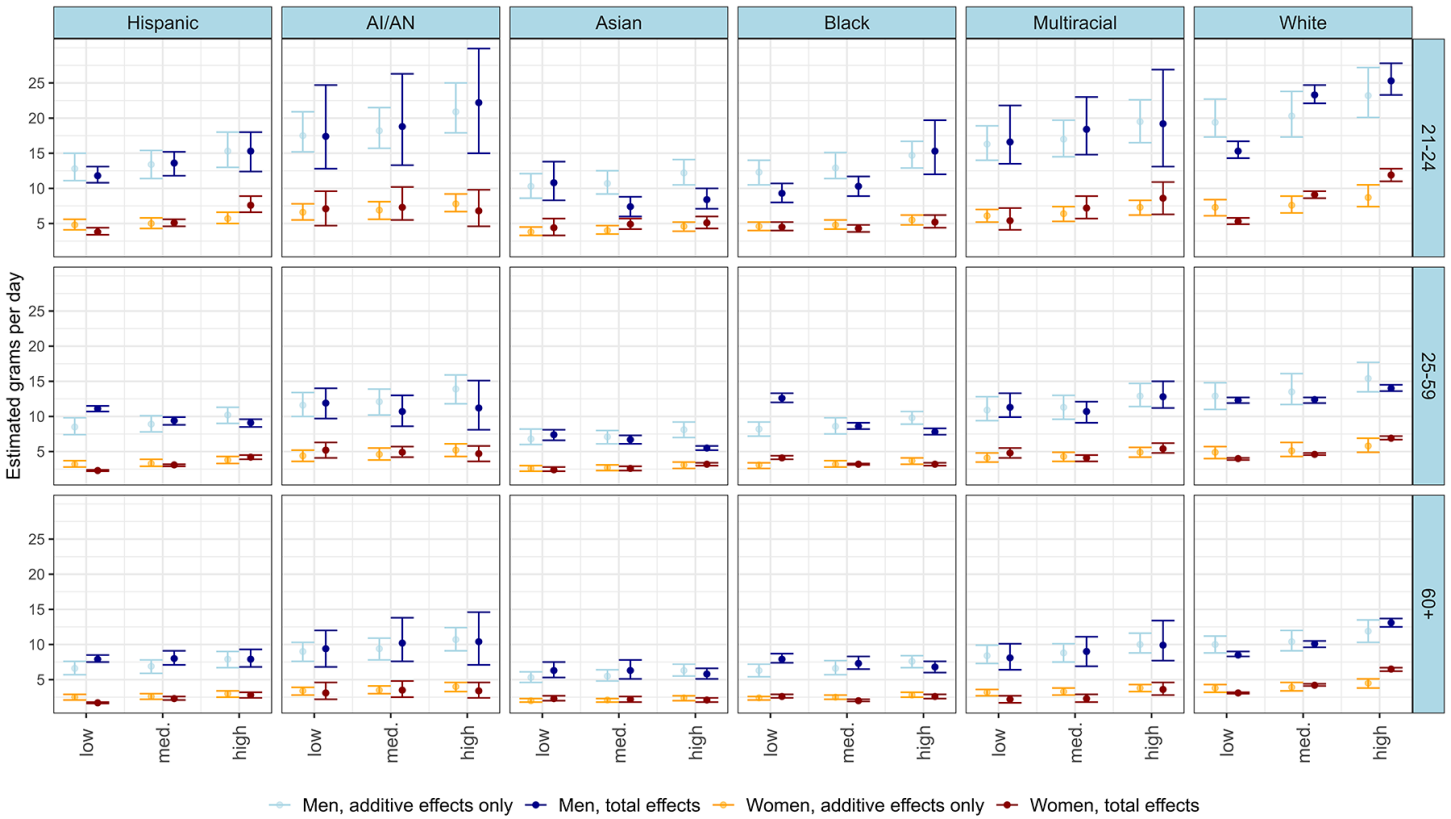
Plot of predicted average alcohol consumption for each intersectional stratum, with error bars indicating 95% credible intervals (comparing predictions based on total effects versus additive effects only).

In Fig. 3, we present the average alcohol consumption of drinkers (in GPD) for each stratum. Again, estimates are provided considering both additive and multiplicative effects, as well as estimates based solely on additive effects. Table 4 supplements this, providing the average consumption for the five strata with i) the highest estimates, ii) the lowest estimates, iii) the largest positive interactions and iv) the largest negative interactions. Predicted GPD for all 108 strata are provided in Supplementary Table 2.

We observed significant variation in the GPD estimates across strata, ranging from 1.7 GPD (less than one drink a week) to 25.3 GPD (~13 standard drinks a week). Similar to the previous findings, we noted the highest consumption levels amongst young individuals with at least some college education, particularly among White, mixed-race, and AI/AN men, and the lowest consumption among older women with a minoritized race and ethnicity. Additionally, the age gradient seen in drinking status is reflected in the average consumption of drinkers, with younger individuals consuming more on average than older individuals, especially among groups with high consumption at a young age.

In other regards, there are notable differences in the pattern of findings between the outcomes. Firstly, the greatest variations in GPD estimates are observed among young men, rather than young women (as was the case for drinking status). The difference between the heaviest and lightest drinking male strata was 19.8 GPD(~10 drinks a week), in contrast to 10.2 GPD (~5 drinks a week) for women.

Secondly, the difference between men and women is more pronounced in relation to GPD, with no female stratum predicted to drink at levels comparable to the highest-consuming men. For example, despite having the highest consumption among women, young White women with high education drank less than half as much as equivalent male strata (11.9 GPD or ~ 6 drinks a week vs 25.3 GPDor ~ 13 drinks a week).

Thirdly, the same educational gradient observed in drinking status is not seen consistently in relation to GPD. While present in several groups, particularly among younger men, it is absent or reversed in others. For example, in the 25–59 age category, Hispanic, Black, and Asian men with high education all have lower predicted GPD than their counterparts with low education.

Significant interaction effects were found for 25 out of the 108 strata. As for drinking status, these interaction effects varied in direction. No patterns in the direction or size of interaction effects were noted, emphasising the complexity of these interaction effects. For example, whilst Black men with low education aged 25–59 exhibited significant positive interaction effects (+4.4 GPD*)*, the same group aged 21–24 exhibited significant negative interaction effects (−3.0 GPD).

### 3.5. Relationship between the proportion of current drinkers and the average consumption of drinkers

**Fig. 4.**
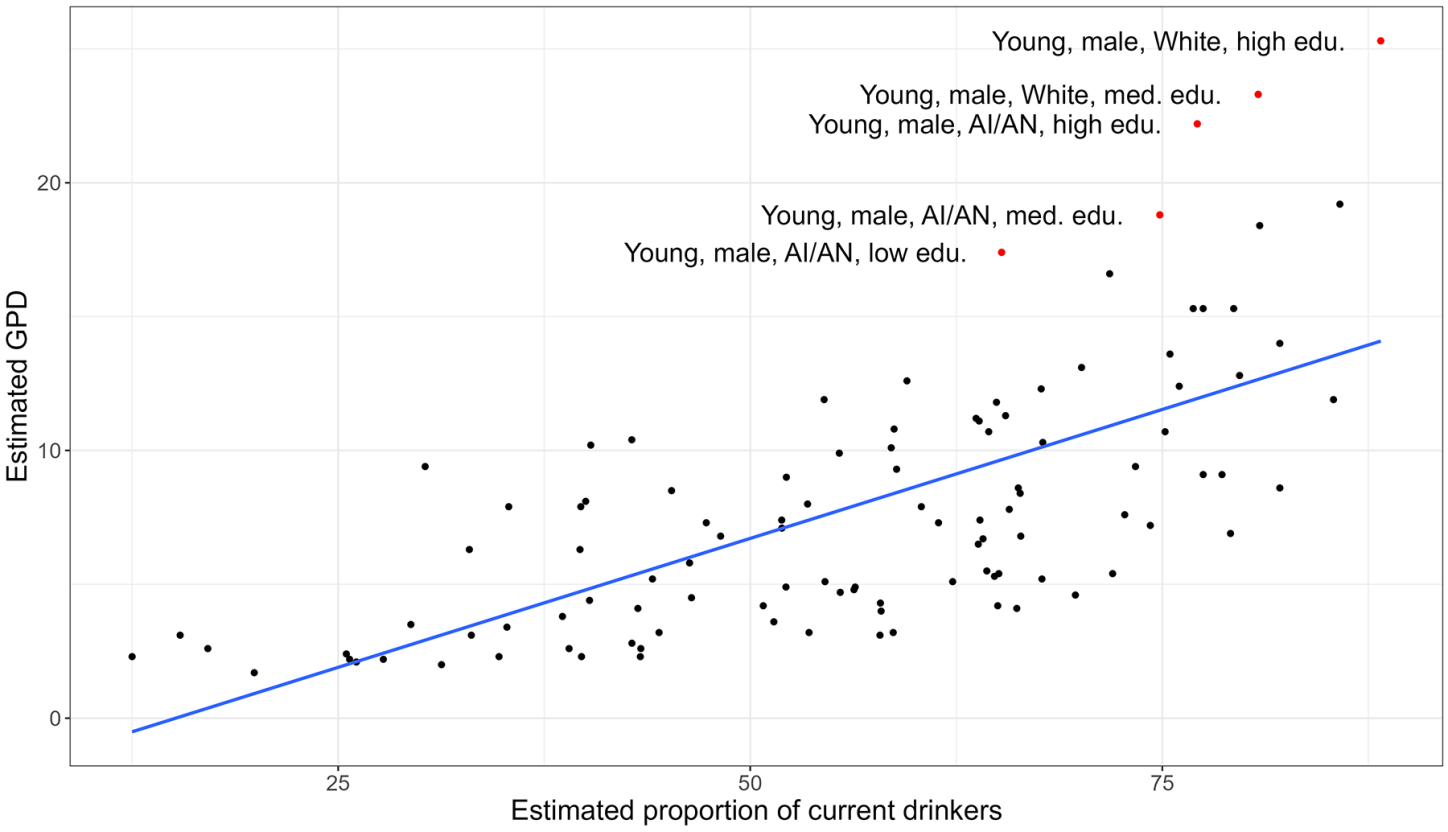
Scatter plot of the correlation between the predicted proportion that are current drinkers and the predicted GPD of drinkers. N = 108 strata. Regression line fitted with OLS regression. Outliers identified in red.

Fig. 4 shows the correlation between the predicted proportion of current drinkers and the predicted daily consumption of drinkers within the same stratum. The correlation coefficient was 0.68, indicating a moderately strong positive linear relationship. We identified five strata as outliers, all of whom were young, White or AI/AN, men. These groups had substantially higher GPD estimates than would be expected based on the proportion of current drinkers in their stratum. For example, young AI/AN men with low education were fairly middling in terms of their predicted proportion of current drinkers (ranked 73rd out of 108 strata; 65.3% current drinkers). Importantly, however, those who *do* drink in this stratum were some of the most heavily drinking (ranked 102nd out of 108; 17.4 GPD).

## 4. Discussion

This study is the first to apply intersectional MAIHDA to understand alcohol consumption disparities, revealing complex patterning that may be missed with traditional regression analyses. Young, highly educated White men were most likely to be drinkers and had the highest average alcohol consumption. Conversely, racially and ethnically minoritized women with lower education, across all age categories, were the least likely to be drinkers and had the lowest average consumption. We identified significant interaction effects for many strata, indicating that simple additive models cannot fully capture intersectional disparities.

Overall, 17% of the variance in the propensity to drink and 12% of the variance in the average consumption of drinkers could be explained by disparities clustered at the between-stratum level. Most of this variance was accounted for by the inclusion of additive main effects but the remaining unexplained variance indicates the presence of interaction effects. The relative importance of the between strata variance for describing individual outcomes is slightly larger than seen in most MAIHDA (typically less than 10% and often less than 5%) (Evans et al., 2024b), though the majority of variance remains at the individual-level. In other words, there is substantial heterogeneity *within* strata and some individuals within a given stratum will have much higher or lower consumption than the group estimate.

We observed greater variation in the proportion of current drinkers among women compared to men. For instance, White women with high education are almost as likely to drink as White men with high education, while Hispanic women with low education are much less likely to drink than Hispanic men with low education. Conversely, the widest spread in the average consumption of drinkers is seen amongst men, with women consistently drinking at much lower levels. The most substantial variation is seen amongst young men, which may indicate that factors influencing consumption vary the most, or have the most differential impact, within this group.

### 4.1. The influence of privilege and disadvantage on drinking

Whilst there are numerous factors that are expected to contribute to these differences in alcohol consumption, including biological, social, psychological, and environmental factors, intersectionality draws attention to the contributions of interlocking social power, oppression, discrimination, and privilege.

Our findings suggest that holding multiple privileged positions may promote heavier drinking. For example, White, young, highly educated men had the highest percentage of current drinkers (88.3%) and consumed the most alcohol on average (25.3 GPD). Interlocking forms of systemic privilege may promote higher levels of drinking among this group by providing greater access to social power and resources, making alcohol consumption more affordable and acceptable, and less harmful. The strong ‘college effect’ seen amongst this group reflects that heavy drinking is often normalized and celebrated among White college students, and a broader cultural norm that perpetuates social dominance and acceptance of risky behaviours within this demographic (Krieger et al., 2018).

Among groups holding a combination of privileged and marginalised positions, we found alcohol consumption to vary. This reflects that low and high-status social positions intersect in complex ways (Bowleg, 2012). For example, level of education has differential influence across intersections. The strong ‘college effect’ observed for White men and women is less pronounced among most other racial and ethnic groups, and even reversed among young Asian, and Black adult, men. Consistently low average consumption amongst young Asian men and women (in comparison to other youth) may be attributed to cultural norms, ethnic identity, and religious affiliations, all of which may serve as protective factors against the dominant subculture of excessive drinking in college (Krieger et al., 2018). Black adult men with high education also drink less than Black adult men with low education, but in contrast to young Asian men, this reversed education trend is driven by relatively *high* consumption amongst those with low education (who have a similar GPD to White adult men with high education). High consumption amongst Black men with low education may reflect drinking to cope with cumulative exposure to a hostile, stressful and discriminatory environment (Banks and Zapolski, 2018; Hatch, 2005), and structural factors such as higher alcohol outlet density in economically deprived, minority neighbourhoods (Lee et al., 2020). In further contrast, we see no significant difference in average consumption by education for AI/AN men and women (as indicated by overlapping CIs). For all AI/AN groups, consumption is relatively high in comparison to other racial and ethnically minoritized groups. Within the AI/AN population, substance use is thought to be inextricably linked to the sociocultural and historical contexts of colonization, historical grief and trauma; specifically, drinking has been reported to be an artifact of colonization, and a means for coping with historical trauma and loss transferred through generations (Gameon and Skewes, 2021; Herron and Venner, 2023).

Our results suggest that holding multiple disadvantaged positions may reduce drinking. Racially and ethnically minoritized (Hispanic, Asian, and Black) women with low or medium education were least likely to be drinkers and drank at the lowest levels, across all age categories. Significant interaction effects sometimes contributed to particularly low consumption. For example, older Hispanic women with low education had a low additive prediction (2.5 GPD) *and* negative interaction terms (−0.8 GPD) resulting in a low overall prediction (1.7 GPD). However, most often these low estimates were due to small additive effects.

Racially and ethnically minoritized women face multiple social disadvantages and geographic inequalities of opportunity (Osypuk and Acevedo-Garcia, 2010) that could be contributing to this pattern. For example, the negative consequences of drinking may be heightened for these groups at work (because of less flexible, less autonomous, and more ‘replaceable’ jobs), at home (for example, if unable to outsource family/homemaking responsibilities), and when going out (for example, due to neighbourhood crime) (Mulia and Bensley, 2020). Further, while drinking has become more socially accepted for White women in privileged classes, women with lower SES, especially racially and ethnically minoritized women, continue to face greater surveillance, stigmatization, and penalization for alcohol use (Schmidt, 2014). Whilst previous literature has found a greater number of alcohol-related social, legal and work consequences for racially minoritized men and *not* for women (Witbrodt et al., 2014), it is plausible that the threat of such consequences may still act as deterrent amongst racially minoritized women. Importantly, although we highlight potential links between social disadvantage and lower alcohol use, women are more susceptible to health harms at the same level of alcohol consumption as men (Erol and Karpyak, 2015). Therefore, from a health perspective, this lower alcohol consumption may beneficial, although the WHO has recently recognised that no level of alcohol consumption is considered safe in relation to human health (WHO, 2023).

Additionally, these groups may have different motivations for, and mechanisms into, (not) drinking. For instance, minoritized groups can derive strength from their shared, minoritized identities through social support and community connectedness, bolstering identity pride, self-esteem and resilience and promoting positive health behaviours (Perrin et al., 2020). In some cases, such as among Black women, low consumption could reflect “drier” cultural norms stemming from, for example, affiliation with religious traditions that eschew alcohol (Herd, 1996).

The nuanced relationship between intersectional social position and alcohol consumption is further emphasised by the presence of significant interaction effects. In some cases, interactions emphasised existing additive patterns and in others they contrasted them. The presence of these significant interaction effects alone does not tell us anything about the relative advantage or disadvantage of a group (Crenshaw, 1989). However, the large number of significant interaction effects highlights the importance of accounting for them. For example, failing to account for interaction effects in relation to alcohol consumption would significantly underestimate the average consumption of Black adult men with low education (12.6 GPD with interaction effects versus 8 GPD without). In turn, this would lead to false conclusions that this group drink less than other racial and ethnic groups of the same age and educational level, where MAIHDA estimates suggest they actually drink *more*. Similarly, typical additive analyses would underestimate the proportion of older White women with high education who are current drinkers (63.8% with interaction effects versus 54.6% without).

The findings of this study underscore the importance of moving beyond single-issue analyses when addressing disparities in alcohol consumption. As highlighted by Crenshaw (1989), systems that are only equipped for such single-issue analyses are likely to overlook the complex realities faced by individuals, who each occupy multiple, intersecting social positions. Broad assumptions like ‘Black men drink less than White men’ and ‘low education groups drink less than higher education groups’ overlook that Black men aged 25–59 with low education drink at similar levels to White men with medium and high education. Similarly, assuming that all young people drink more than older people overlooks that for Black men with low education, those aged 25–59 drink *more* than those aged 21–24. For all intersections, it is critical to note that there is also substantial heterogeneity *within* strata. For example, high levels of drinking are still seen within subgroups of the Asian population (Cook et al., 2012), and there are many *non*-drinkers within the AI/AN population, despite high average consumption amongst drinkers (Herman-Stahl and Chong, 2002).

This study has several implications for research, policy, and critical praxis. Firstly, it identifies several specific understudied groups who require further attention due to high consumption levels and/or higher consumption than would be expected based on additive only effects (e. g., young AI/AN and mixed-race men, adult Black men with low education, older White women with high education). Further work with these specific intersections is needed, such as qualitative research and analytical analyses, to help understand why these nuanced differences exist, the causal pathways underpinning them, and to identify targetable policy/intervention areas. Further, at present there is a sparsity of studies addressing alcohol policy effects by sex, SES, and race and ethnicity, reflecting a major research gap (Kilian et al., 2023). This study emphasises, that not only should policy evaluations consider these factors, but that appropriate granularity is required (e.g., not grouping AI/AN and Asian together) and that ideally differential impact should be assessed across intersections.

It is critical to note that the groups who we identified as consuming the most alcohol are not necessarily those who experience the greatest alcohol-related harms. There is strong evidence of an ‘alcohol harm paradox’ in the US, where individuals with low education suffer higher alcohol-attributable mortality despite consuming the same, or lower amounts of alcohol (Probst et al., 2021), with similar paradoxical relationships also found in relation to race and ethnicity (Zapolski et al., 2014). Many explanations have been proposed for this, including biology, behaviour, environmental factors, life course disadvantage, access to health care, policy and other upstream factors, and methodological issues; however, most have received little empirical testing and the paradox remains poorly understood (Boyd et al., 2022). This study reveals that accounting for interactions results in higher estimates of total consumption for certain subgroups and lower estimates for others. In the latter case, this could indicate that findings of differential vulnerability (i.e., drinking the same amount of alcohol leads to more harm than expected) may be greater than previously predicted for some subgroups (Mulia and Zemore, 2012). Future research mapping the intersectional patterning of alcohol *harms* would complement this study and help to identify where to target resources, interventions, and policies to best address inequities in alcohol-attributable harms. Ignoring intersectional patterning may lead to a widening of health inequalities if only high-risk, and not high vulnerability, groups are considered.

In addition to increasing our understanding of alcohol consumption patterns, this analysis shows how MAIHDA can be applied to heavily positively skewed data, whilst still appropriately accounting for residuals. This approach may be used to explore other typically skewed outcomes, including other health behaviours (smoking, drug use, physical activity levels etc.), disease severity, and health related costs.

### 4.2. Limitations

This study has several limitations. First, this study is exploratory and descriptive, rather than analytical, and uses social positions as an indicator of exposure to a shared social context. As a result, this study risks reinforcing perceptions of entrenched disparities, or unintentionally stigmatizing certain groups, particularly given alcohol use disorder is itself highly stigmatized (Kilian et al., 2021). We therefore urge readers to view inter-group consumption differences as reflecting social context, not inherent differences, and present these descriptive findings as a foundation for future analytical and structural research.

Second, alternative intersectional group classifications could have offered different insights. Other variables associated with alcohol consumption, such as sexual orientation, religious affiliation, and gender (rather than sex), may have provided important insights, however, were not available. There were other relevant variables available in the data that we could have included (such as nativity status). However, as more categories are added, the more likely that some intersectional groups will contain few or no individuals. Additionally, there is a substantial increase in strata number with even a single social category addition, making the interpretation and presentation of findings more challenging. Race and ethnicity were based on closed response questions, which may not fully align with individuals’ identities, and the educational categories used may reflect slightly different things at different ages, given educational status stabilises with age. Additionally, the NHIS excludes institutionalized populations, such as those in nursing homes or prisons. Since a key element of intersectionality is highlighting often invisible populations, addressing this limitation in future work, such as through dedicated primary data collection studies, is crucial.

Third, we focused on average consumption rather than drinking patterns. While we account for HED within our measure of GPD, a specific HED analysis (in progress) may provide different insights. Additionally, self-reported alcohol consumption can be subject to social desirability bias (Davis et al., 2010), which may vary by sociodemographic position. For example, groups facing ‘stereotype threat’ in relation to alcohol may underreport to avoid perpetuating stereotypes (Green et al., 2007). If social desirability bias varies across intersectional groups, this may have biased our results.

Fourth, we chose to pool data across multiple years to facilitate adequate subgroup sample sizes, given the large number of intersectional strata under consideration. However, we note that alcohol trends have changed over time, with period and cohort effects observed for some groups. For example, there have been recent increases in consumption and heavy drinking among women and older ages (Kerr et al., 2024), and ‘baby boomer’ and ‘generation X’ cohorts have been shown to drink significantly more compared to their earlier/later counterparts (Keyes, 2022). Further, US demographics have shifted over time for particular racialized groups. For example, the number of foreign-born (versus US born) Hispanics, and the countries of origin for those foreign-born, have shifted. We do not know what the net effect of these shifts might be on the average consumption of Hispanic young adults in our sample. As a robustness check, we ran a sensitivity analysis, restricting the sample to the years 2010–2018 and re-estimating average GPD (see Appendix A, Supplemental Fig. 3). In this more recent time period, CIs were wider (as expected due to the smaller sample size) and there was no longer a significant education gradient seen for 25–59-year-old Hispanic men, or older White women. However, there were otherwise no major differences between the two samples.

Finally, while the NHIS contains sample weights to adjust for the probability of selection and non-response, MCMC methods don’t generally accommodate survey weights, so our results reflect unweighted estimates. Given the disaggregated nature of MAIHDA analyses and our inclusion of age, race and ethnicity, and sex within intersectional strata, we do not expect that the use of subject weights would have significant impact on the findings.

## 5. Conclusion

This study used quantitative intersectional methods to map out alcohol consumption in the US, incorporating both additive and multiplicative effects. We identified significant intersectional disparities in the proportion of current drinkers and the average consumption of those drinkers, which could not be fully explained by additive effects alone. Our findings reveal how interlocking social and environmental factors, including power, privilege, discrimination, and culture, produce disparities in alcohol consumption – emphasising that sociodemographic factors should not be considered in isolation when researching or addressing alcohol consumption. Further work is needed to understand the relationship between consumption and harm across intersections and to identify the drivers of differential vulnerability, where relevant.

## Supplementary Material

Table S1

Appendix B

Table S2

Appendix A

Supplementary material can be found online at https://doi.org/10.1016/j.socscimed.2024.117514.

## Figures and Tables

**Table 1.** Characteristics of analytic sample adults, taken from the National Health Interview Survey, 2010–2018.

	Full sample		Current drinkers	
	n	%	n	%
**Total**	526,557	-	327,016	-
**Age**				
21–24	33,280	6.3	23,921	7.3
25–59	334,664	63.6	227,084	69.4
60+	158,613	30.1	76,011	23.2
**Sex**				
Men	229,761	43.6	159,122	48.7
Women	296,796	56.4	167,894	51.3
**Race and ethnicity**				
White	334,883	63.6	225,651	69.0
Black	72,074	13.7	37,099	11.3
Asian	24,674	4.7	12,193	3.7
Multiracial	6,727	1.3	4,462	1.4
AI/AN	3,093	0.6	1,601	0.5
Hispanic	85,106	16.2	46,010	14.1
**Education**				
High school or less	226,287	43.0	114,014	34.9
Some college	153,095	29.1	102,728	31.4
4+ years college	147,175	28.0	110,274	33.7
**Outcomes**	mean	(SD)	mean	(SD)
GPD	4.9	12.0	7.9	14.4

**Table 2:** Parameter estimates for logistic models of drinking status (drinker/non-drinker) and linear models of average consumption (GPD).

	Drinking status (logistic models)		GPD (linear models)	
	Null model	Main effects model	Null model	Main effects model
	Estimate (95% CI)	Estimate (95% CI)	Estimate (95% CI)	Estimate (95% CI)
**Fixed effects: Regression Coefficients**				
Intercept	0.26 (0.15, 0.34)	1.21 (1,15, 1.29)	0.57 (0.43, 0.66)	1.62 (1.51, 1.76)
Sex				
Women	–	−0.55 (−0.61, −0.48)	–	−0.98 (−1.07, −0.91)
Age				
25–59	–	−0.38 (−0.47, −0.28)	–	−0.41 (−0.65, −0.31)
60+	–	−1.30 (−1.39, −1.17)	–	−0.66 (−0.78, −0.55)
Race and ethnicity				
Hispanic	–	−0.77 (−0.85, −0.66)	–	−0.46 (−0.61, −0.31)
Black	–	−1.06 (−1.17, −0.95)	–	−0.64 (−0.81, −0.48)
Asian	–	−0.22 (−0.37, −0.08)	–	−0.18 (−0.35, 0.01)
Mixed-race	–	−0.80 (−0.98, −0.64)	–	−0.11 (−0.29, 0.05)
AI/AN	–	−0.55 (−0.65, −0.43)	–	−0.41 (−0.56, −0.26)
Education				
Some college	–	0.57 (0.49, 0.66)	–	0.04 (−0.07, 0.14)
4+ years college	–	0.82 (0.67, 0.91)	–	0.18 (0.08, 0.26)
**Random effects: Variances**				
Stratum-Level	0.67 (0.51, 0.84)	0.05 (0.04, 0.07)	0.41 (0.32, 0.51)	0.04 (0.03, 0.06)
Individual-Level	3.29	3.29	2.98 (2.96, 2.99)	2.98 (2.96, 3.00)
**Summary Statistics**				
VPC	16.8%	1.4%	12.0%	1.4%
PCV	–	91.6%	–	88.7%

Reference categories: Men; 21–24; White; High school or less. All groups except Hispanic are non-Hispanic e.g., Black = Non-Hispanic Black.

CI = Credible Intervals, VPC = Variance Partition Coefficient, PCV = Proportional Change in Variation.

Strata: n = 108; Individuals: n = 526,557 (for drinking status), n = 327,016 (for grams per day).

**Table 3:** Predicted % of current drinkers (and 95% CI) for the five strata with the highest predicted values, lowest predicted values, largest positive interactions, and largest negative interactions.

	Total			Additive only			Interaction		
	%	L	U	%	L	U	%	L	U
**Five strata with highest estimates**									
M, 21–24, White, high	88.3	86.8	89.8	88.4	87.3	89.3	−0.1	−1.8	1.7
M, 21–24, Mixed-race, high	85.8	81.3	90.0	86.0	83.8	88.0	−0.2	−5.6	3.9
F, 21–24, White, high	85.4	84.1	86.7	81.5	79.6	82.9	**3.9**	**1.9**	**6.2**
F, 21–24, Mixed-race, high	82.1	77.2	86.7	78.0	73.8	81.0	4.1	0.0	8.5
M, 25–59, White, high	82.1	81.6	82.6	83.9	81.9	85.8	−1.8	−3.6	0.2
**Five strata with largest significant, positive interactions (higher % drinkers than expected** ^a^ **)**									
F, 60+, White, high	63.8	62.8	64.8	54.6	51.7	57.0	**9.2**	**6.6**	**12.3**
M, 60+, Asian, low	33.0	29.7	36.0	24.1	21.7	26.7	**8.9**	**4.5**	**12.8**
M, 25–59, Black, low	59.5	58.1	60.6	51.8	48.6	55.9	**7.7**	**3.5**	**10.9**
M, 25–59, Asian, low	51.9	49.7	54.1	44.4	41.4	47.6	**7.5**	**4.3**	**11.6**
M, 25–59, Hispanic, low	63.9	63.0	64.7	57.2	52.8	60.1	**6.7**	**3.5**	**10.7**
**Five strata with lowest estimates**									
F, 60+, Asian, low	12.5	11.0	14.4	15.5	13.9	17.3	**−3.0**	**−5.1**	**−0.7**
F, 60+, AI/AN, low	15.4	12.3	19.3	19.3	16.8	22.1	**−3.8**	**−7.6**	**−0.7**
F, 60+, Black, low	17.1	16.3	18.0	19.8	17.6	21.5	**−2.7**	**−4.8**	**−0.2**
F, 60+, Hispanic, low	19.9	18.9	20.8	23.5	21.5	25.2	**−3.6**	**−5.8**	**−1.3**
F, 25–59, Asian, low	25.5	24.0	27.3	31.6	29.0	34.6	**−6.1**	**−8.6**	**−3.2**
**Five strata with largest significant, negative interactions (lower % drinkers than expected** ^a^ **)**									
F, 21–24, Hispanic, low	38.6	36.9	40.5	52.9	50.5	55.3	**−14.3**	**−17.3**	**−11.5**
F, 25–59, Hispanic, low	34.8	33.9	35.5	43.6	39.4	46.6	**−8.8**	**−11.6**	**−4.9**
M, 60+, Mixed-race, high	55.4	50.2	60.9	62.6	59.3	66.0	**−7.2**	**−13.7**	**−0.8**
F, 25–59, Asian, high	44.5	42.9	45.8	51.2	47.1	54.3	**−6.7**	**−9.7**	**−2.4**
M, 25–59, AI/AN, high	63.7	58.3	69.6	70.1	65.5	74.0	**−6.4**	**−11.8**	**−0.9**

Predictions made for the year 2009 for all strata.

Interaction effects with 95% CIs excluding 0 are shown in bold.

% = Predicted % of current drinkers, L = Lower bound of credible interval, U = Upper bound of credible interval.

M = Male, F= Female, low = high school or less, high = 4+ years college.

a In reference to what would be expected based on additive effects only.

**Table 4:** Predicted GPD (and 95% CI) for the five strata with the highest estimates, lowest estimates, largest positive interactions, and largest negative interactions.

	Total			Additive only			Interaction only		
	est.	L	U	est.	L	U	est.	L	U
**Five strata with highest estimates**									
M, 21–24, White, high	25.3	23.3	27.8	23.2	20.1	27.2	2.2	−1.4	6.2
M, 21–24, White, medium	23.3	22.1	24.7	20.3	17.3	23.8	3.0	−0.6	6.1
M, 21–24, AI/AN, high	22.2	15	29.9	20.9	17.9	25	1.4	−5.8	8.5
M, 21–24, multiracial, high	19.2	13.1	26.9	19.5	16.5	22.6	−0.2	−5.8	8.3
M, 21–24, AI/AN, medium	18.8	13.3	26.3	18.2	15.7	21.5	0.5	−5.1	8.7
**Strata with largest positive & significant interactions (drink more than would be expected** ^a^ **)**									
M, 25–59, Black, low	12.6	12	13.3	8.2	7.2	9.2	**4.4**	**3.2**	**5.7**
F, 21–24, White, high	11.9	11	12.8	8.7	7.4	10.5	**3.2**	**1.4**	**4.7**
M, 25–59, Hispanic, low	11.1	10.7	11.5	8.5	7.4	9.8	**2.5**	**1.5**	**3.6**
F, 60+, White, high	6.5	6.2	6.7	4.5	3.8	5.1	**2.0**	**1.4**	**2.7**
F, 21–24, Hispanic, high	7.6	6.6	8.9	5.7	5.0	6.6	**1.9**	**0.5**	**3.2**
**Five strata with lowest estimates**									
F, 60+, Hispanic, low	1.7	1.6	1.8	2.5	2.1	2.9	**−0.8**	**−1.3**	**−0.5**
F, 60+, NH Black, medium	2.0	1.9	2.2	2.5	2.2	2.8	**−0.5**	**−0.8**	**−0.1**
F, 60+, NH Asian, high	2.1	1.8	2.4	2.4	2.0	2.7	−0.3	−0.6	0.1
F, 60+, NH Asian, medium	2.2	1.8	2.6	2.1	1.8	2.3	0.1	−0.4	0.6
F, 60+, multiracial, low	2.2	1.7	2.7	3.2	2.7	3.6	**−1.0**	**−1.6**	**−0.4**
**Strata with largest negative & significant interactions (drink less than would be expected** ^a^ **)**									
M, 21–24, White, low	15.3	14.3	16.7	19.4	17.3	22.7	**−4.2**	**−7.4**	**−1.3**
M, 21–24, Asian, high	8.4	7.1	10	12.2	10.5	14.1	**−3.8**	**−6**	**−1.4**
M, 21–24, Asian, med	7.4	6	8.8	10.7	9.2	12.5	**−3.3**	**−4.9**	**−1.5**
M, 21–24, Black, low	9.3	8	10.7	12.3	10.5	14	**−3**	**−5.2**	**−0.9**
M, 25–59, Asian, high	5.5	5.2	5.8	8.1	7	9.2	**−2.7**	**−3.6**	**−1.5**

Predictions made for the year 2009 for all strata.

Interaction effects with 95% CIs excluding 0 are shown in bold.

E = Estimate, L = Lower bound of credible interval, U = Upper bound of credible interval.

M = Male, F= Female, low = high school or less, medium = some college, high = 4+ years college.

a In reference to what would be expected based on additive effects only.

## Data Availability

The authors do not have permission to share the data however it is publically available. The code to reproduce the analysis is publically available on Github (details in manuscript).
