# Supplementary material for: An analysis of intersectional disparities in alcohol consumption in the US
Source: Soc Sci Med. Author manuscript; Available in PMC 2026 Jan 30. (PMC12857125; doi:10.1016/j.socscimed.2024.117514)
Supplement: Appendix B [file NIHMS2131773-supplement-Appendix_B.docx]

**Appendix B. Supplementary material – statistical details**

**1. Approach for assigning GPD to individuals**

For individuals who reported drinking less than 5 drinks on a typical drinking day, but sometimes drinking more than 5 (i.e., irregular heavy episodic drinkers), we used an expanded quantity/frequency approach, to account for increased consumption on heavy drinking days. For all others we employed a basic quantity/frequency approach. In all calculations, we assumed a ‘standard drink’ contained 14 grams of alcohol.

*Basic quantity/frequency approach:*

$$\mathrm{gpd}=14\times\frac{drinks per day\times number of drinking days}{365}$$

*Expanded quantity/frequency approach:*

For individuals reporting irregular heavy episodic drinking, we used an expanded quantity/frequency approach, to account for the increase in consumption on HED days. We allocated a conservative estimate of 5 drinks on HED days.

$$\mathrm{gpd}=14\times\frac{drinks per day \times\left( drinking days -HED days \right)+5\times HED days}{365}$$

**2. MAIHDA models**

The proportion of current drinkers in each stratum was estimated using logistic MAIHDA models and was represented as follows:

Null model

$$y_{ij}\sim\mathrm{Binomial}\left( 1,\pi_{j} \right)$$

$$\mathrm{logit}\left( \pi_{j} \right)\equiv\log\left( \frac{\pi_{j}}{1-\pi_{j}} \right)=\beta_{0}+u_{j}$$

$$u_{j}\sim N\left( 0,\sigma_{u}^{2} \right)$$

Main effects model

$$\mathrm{logit}\left( \pi_{j} \right)\equiv\log\left( \frac{\pi_{j}}{1-\pi_{j}} \right)=\beta_{0}+\beta_{1}x_{1j}\ldots+\beta_{p}x_{pj}+u_{j}$$

$$u_{j}\sim N\left( 0,\sigma_{u}^{2} \right)$$

where

- $y_{ij}$ denotes whether an individual is a current drinker (1) or not (0), for individual 𝑖 in stratum 𝑗.
- $\pi_{j}$ denotes the probability of being a current drinker for all individuals in stratum $j$.
- $\beta_{0}$ denotes the intercept.

- $u_{j}$ (in null model): represents the stratum random effect (the difference between the intersectional group estimates and the grand mean) on the logit scale.
- $\beta_{1},\ldots,\beta_{p}$ represent the main (fixed) effects of social categories 1 through $p$, defined by dummy variables $x_{1j},\ldots,x_{pj}$ .
- $u_{j}$ (in main effects model): represents the stratum random effect (the difference between the intersectional group estimates and the expected estimate for that intersection on the logit scale based on the main additive effects only).

We estimated the average alcohol consumption of current drinkers using linear MAIHDA models, where the models for $\ln\left( y_{ij} \right)$, the log transformed version of the positively skewed outcome $y_{ij}$, can be written as:

Null model

$$\ln\left( y_{ij} \right)=\beta_{0}+u_{j}+e_{ij}$$

$$u_{j}\sim N\left( 0,\sigma_{u}^{2} \right)$$

$$e_{ij}\sim N\left( 0,\sigma_{e}^{2} \right)$$

Main effects model

$$\ln\left( y_{ij} \right)=\beta_{0}+\beta_{1}x_{1j} \ldots+\beta_{p}x_{pj}+u_{j}+e_{ij}$$

$$u_{j}\sim N\left( 0,\sigma_{u}^{2} \right)$$

$$e_{ij}\sim N\left( 0,\sigma_{e}^{2} \right)$$

where

- $y_{ij}$ now represents the GPD for individual 𝑖 in stratum 𝑗.
- $e_{ij}$ represents the within-stratum random residual at the individual level.

**3. Modelling a log-transformed outcome in a two-level random-intercept model**

# The model

Consider a two-level random-intercept model for $\ln\left( y_{ij} \right)$, the log transformed version of positively skewed outcome $y_{ij}$, and one covariate $x_{j}$. The model can be written as

$$\ln\left( y_{ij} \right)=\beta_{0}+\beta_{1}x_{j}+u_{j}+e_{ij}$$

$$u_{j}\sim N\left( 0,\sigma_{u}^{2} \right)$$

$$e_{ij}\sim N\left( 0,\sigma_{e}^{2} \right)$$

# Expected untransformed outcome, conditional on $\boldsymbol{x}_{\boldsymbol{j}}$ and $\boldsymbol{u}_{\boldsymbol{j}}$

When calculating model predictions, the quantity of interest is the expected untransformed outcome, conditional on $x_{j}$ and $u_{j}$, $E\left( y_{ij} | x_{j},u_{j} \right)$. This can be obtained by averaging the outcome at each value of $x_{j}$ and $u_{j}$ over the distribution of $e_{ij}$.

## What to do

To obtain this quantity, we first rewrite the model in terms of the untransformed outcome $y_{ij}$

$$y_{ij}=\exp\left( \beta_{0}+\beta_{1}x_{j}+u_{j}+e_{ij} \right)$$

$$=\exp\left( \beta_{0}+\beta_{1}x_{j}+u_{j} \right)\exp\left( e_{ij} \right)$$

where $\exp\left( e_{ij} \right)$ is log normally distributed meaning that if we were to apply a log transform to $\exp\left( e_{ij} \right)$ this would result in a normally distributed variable, namely $e_{ij}$.

The expected untransformed outcome $E\left( y_{ij} | x_{j},u_{j} \right)$ is then given by

$$E\left( y_{ij} | x_{j},u_{j} \right)=\exp\left( \beta_{0}+\beta_{1}x_{j}+u_{j} \right)E\left\{ \exp\left( e_{ij} \right) | x_{j},u_{j} \right\}$$

$$=\exp\left( \beta_{0}+\beta_{1}x_{j}+u_{j} \right)\exp\left( \frac{\sigma_{e}^{2}}{2} \right)$$

where we have made use of mathematical result that the expectation of a log normally distributed variable is equal to the exponential of half the variance of its log transformed version.

## What not to do

When calculating model predictions, many researchers forget to multiply by $\exp\left( \frac{\sigma_{e}^{2}}{2} \right)$. This is because they first calculate the mean of the log of the outcome

$$E\left\{ \ln\left( y_{ij} \right) | x_{j},u_{j} \right\}=\beta_{0}+\beta_{1}x_{j}+u_{j}$$

Then they calculate the exponential of this quantity and assume that this gives the mean of the outcome

$$\exp\left( E\left\{ \ln\left( y_{ij} \right) | x_{j},u_{j} \right\} \right)=\exp\left( \beta_{0}+\beta_{1}x_{j}+u_{j} \right)$$

But, this is not true. The mean of the outcome does not equal the exponential of the mean of the log of the outcome.

$$E\left( y_{ij} | x_{j},u_{j} \right)\neq\exp\left( E\left\{ \ln\left( y_{ij} \right) | x_{j},u_{j} \right\} \right)$$

# Expected untransformed outcome, conditional on on $\boldsymbol{x}_{\boldsymbol{j}}$

When calculating model predictions, another quantity of interest is the expected untransformed outcome, conditional on $x_{j}$, $E\left( y_{ij} | x_{j} \right)$. This can be obtained by averaging the outcome at each value of $x_{j}$ over the distributions of both $u_{j}$ and $e_{ij}$.

## What to do

To obtain this quantity, we first rewrite the model for the untransformed outcome $y_{ij}$

$$y_{ij}=\exp\left( \beta_{0}+\beta_{1}x_{j}+u_{j}+e_{ij} \right)$$

$$=\exp\left( \beta_{0}+\beta_{1}x_{j} \right)\exp\left( u_{j} \right)\exp\left( e_{ij} \right)$$

The expected untransformed outcome, conditional on $x_{j}$ is given by

$$E\left( y_{ij} | x_{j} \right)=\exp\left( \beta_{0}+\beta_{1}x_{j} \right)E\left\{ \exp\left( u_{j} \right) | x_{j} \right\}E\left\{ \exp\left( e_{ij} \right) | x_{j} \right\}$$

$$=\exp\left( \beta_{0}+\beta_{1}x_{j} \right)\exp\left( \frac{\sigma_{u}^{2}}{2} \right)\exp\left( \frac{\sigma_{e}^{2}}{2} \right)$$

## What not to do

When calculating model predictions, many researchers forget to multiply by $\exp\left( \frac{\sigma_{u}^{2}}{2} \right)\exp\left( \frac{\sigma_{e}^{2}}{2} \right)$. This is because they first calculate the mean of the log of the outcome

$$E\left\{ \ln\left( y_{ij} \right) | x_{j} \right\}=\beta_{0}+\beta_{1}x_{j}$$

Then they calculate the exponential of this quantity and assume that this gives the mean of the outcome

$$\exp\left( E\left\{ \ln\left( y_{ij} \right) | x_{j} \right\} \right)=\exp\left( \beta_{0}+\beta_{1}x_{j} \right)$$

But, this is not true. The mean of the outcome does not equal the exponential of the mean of the log of the outcome.

$$E\left( y_{ij} | x_{j} \right)\neq\exp\left( E\left\{ \ln\left( y_{ij} \right) | x_{j} \right\} \right)$$

# Difference in expected untransformed outcome attributable to the $\boldsymbol{u}_{\boldsymbol{j}}$

So far we have discussed the expected untransformed outcome conditional on $x_{j}$ and $u_{j}$, $E\left( y_{ij} | x_{j},\boldsymbol{u}_{\boldsymbol{j}} \right)$ and the expected untransformed outcome conditional only on $x_{j}$, $E\left( y_{ij} | x_{j} \right)$. We can then calculate the difference between these two quantities as a measure of the contribution of $\boldsymbol{u}_{\boldsymbol{j}}$ to $E\left( y_{ij} | x_{j},\boldsymbol{u}_{\boldsymbol{j}} \right)$.

#

$$\boldsymbol{D}_{\boldsymbol{j}}\boldsymbol{=}\mathbf{E}\left( \boldsymbol{y}_{\boldsymbol{ij}} | \boldsymbol{x}_{\boldsymbol{j}}\boldsymbol{,}\boldsymbol{u}_{\boldsymbol{j}} \right)\boldsymbol{-}\mathbf{E}\left( \boldsymbol{y}_{\boldsymbol{ij}} | \boldsymbol{x}_{\boldsymbol{j}} \right)\boldsymbol{=}\exp\left( \boldsymbol{\beta}_{\boldsymbol{0}}\boldsymbol{+}\boldsymbol{\beta}_{\boldsymbol{1}}\boldsymbol{x}_{\boldsymbol{j}} \right)\exp\left( \frac{\boldsymbol{\sigma}_{\boldsymbol{e}}^{\boldsymbol{2}}}{\boldsymbol{2}} \right)\left\{ \exp\left( \boldsymbol{u}_{\boldsymbol{j}} \right)\boldsymbol{-}\exp\left( \frac{\boldsymbol{\sigma}_{\boldsymbol{u}}^{\boldsymbol{2}}}{\boldsymbol{2}} \right) \right\}$$

# Alternative approach when $\boldsymbol{e}_{\boldsymbol{ij}}$ are not normally distributed

The above use of

$$E\left\{ \exp\left( e_{ij} \right) | x_{j},u_{j} \right\}=\exp\left( \frac{\sigma_{e}^{2}}{2} \right)$$

assumes that the residuals $e_{ij}$ are normally distributed and therefore that $\exp\left( e_{ij} \right)$ are log normally distributed. If $e_{ij}$ are not normally distributed, then it would be preferable to integrate out the $\exp\left( e_{ij} \right)$ using their actual distribution rather than naively assume that they are log normally distributed. This can be done via a three-step approach. In the first step, we obtain an estimate for $e_{ij}$ defined as

$$\hat{e}_{ij}=\ln\left( y_{ij} \right)-\left( \hat{\beta}_{0}+\hat{\beta}_{1}x_{j}+\tilde{u}_{j} \right)$$

where $\tilde{u}_{j}$ is the predicted stratum random effect. In the second step, we obtain an estimate for $E\left\{ \exp\left( e_{ij} \right) | x_{j},u_{j} \right\}$ defined as

$$\frac{1}{N}\sum_{j=J}^{J} \sum_{i=1}^{n_{j}} \exp(\hat{e}_{ij})$$

where $N=\sum_{j=J}^{J} n_{j}$ is the total number of individuals across all strata. In the third step, for each $j$, we compute the predicted values as

$$\hat{y}_{j}=\exp\left( \hat{\beta}_{0}+\hat{\beta}_{1}x_{j}+\tilde{u}_{j} \right)\frac{1}{N}\sum_{j=J}^{J} \sum_{i=1}^{n_{j}} \exp(\hat{e}_{ij})$$

George Leckie

17/05/2024

**4. Calculating VPC and PCV**

VPC and PCV were calculated as follows:

$$\mathrm{VPC}= \frac{\sigma_{u}^{2}}{\sigma_{u}^{2}+ \sigma_{e}^{2}}$$

$$\mathrm{PCV}= \frac{\sigma_{u}^{2}\left( null model \right)- \sigma_{u}^{2}(full model)}{\sigma_{u}^{2}(null model)}$$

**5. MCMC estimation procedure**

All models were fit using Bayesian Markov Chain Monte Carlo (MCMC) estimation procedures with ‘diffuse’ or ‘flat’ prior belief distributions and IGLS starting values (MLwiN defaults). The models were run for a burn-in period of 5000 iterations, followed by 5000 main iterations. A thinning factor of 50 was used, meaning that every 50th sample from the MCMC chain was used to inform the posterior distribution^52^. Model convergence was assessed based on the MLwiN default convergence tolerance of 10-2 and visual inspection.

The outcome of interest was calculated for each iteration of the MCMC chains, yielding an MCMC chain for the outcome. The mean of this chain represents the point estimate, with the 2.5th and 97.5th percentiles providing the bounds of the 95% CI. This method was used to predict CIs around the total estimates, including both 'additive only' and 'interaction only' effects.

Analyses were conducted in R 4.2.2 using the R2MLwiN package (version ‘0.8.8’) ^53^.
