# Supplementary material for: An analysis of intersectional disparities in alcohol consumption in the US
Source: Soc Sci Med. Author manuscript; Available in PMC 2026 Jan 30. (PMC12857125; doi:10.1016/j.socscimed.2024.117514)
Supplement: Appendix A [file NIHMS2131773-supplement-Appendix_A.docx]

**Supplementary tables and figures**

- Supplementary tables 1 & 2 provided as separate excel files.

| **Supplementary table 3. Model coefficients and variance (drinking status)** | | | | | | |
| --- | --- | --- | --- | --- | --- | --- |
|  | **Null model** | | | **Main effects model** | | |
|  | **est.** | **lower CI** | **upper CI** | **est.** | **lower CI** | **upper CI** |
| **Fixed effects: Regression Coefficients** | | | | | | |
| **Intercept** | 0.26 | 0.15 | 0.34 | 1.21 | 1.15 | 1.29 |
| **Sex/ gender** |  |  |  |  |  |  |
| Female | - | - | - | -0.55 | -0.61 | -0.48 |
| **Age** |  |  |  |  |  |  |
| 25-59 | - | - | - | -0.38 | -0.47 | -0.28 |
| 60+ | - | - | - | -1.30 | -1.39 | -1.17 |
| **Race and ethnicity** |  |  |  |  |  |  |
| Hispanic | - | - | - | -0.77 | -0.85 | -0.66 |
| NH Black | - | - | - | -1.06 | -1.17 | -0.95 |
| NH Asian | - | - | - | -0.22 | -0.37 | -0.08 |
| NH Multiracial | - | - | - | -0.80 | -0.98 | -0.64 |
| NH AI/AN | - | - | - | -0.55 | -0.65 | -0.43 |
| **Education** |  |  |  |  |  |  |
| Some college | - | - | - | 0.57 | 0.49 | 0.66 |
| 4+ years college | - | - | - | 0.82 | 0.67 | 0.91 |
| **Survey year** |  |  |  |  |  |  |
| 2001 | 0.04 | 0.01 | 0.07 | 0.04 | 0.00 | 0.07 |
| 2002 | 0.02 | -0.01 | 0.06 | 0.02 | -0.02 | 0.05 |
| 2003 | -0.02 | -0.06 | 0.01 | -0.03 | -0.07 | 0.00 |
| 2004 | -0.01 | -0.05 | 0.03 | -0.01 | -0.05 | 0.02 |
| 2005 | -0.01 | -0.04 | 0.03 | -0.01 | -0.04 | 0.03 |
| 2006 | -0.01 | -0.05 | 0.03 | -0.01 | -0.05 | 0.02 |
| 2007 | 0.03 | -0.01 | 0.07 | 0.02 | -0.02 | 0.06 |
| 2008 | 0.15 | 0.12 | 0.19 | 0.15 | 0.10 | 0.19 |
| 2009 | 0.19 | 0.16 | 0.23 | 0.19 | 0.15 | 0.22 |
| 2010 | 0.18 | 0.14 | 0.23 | 0.18 | 0.15 | 0.21 |
| 2011 | 0.21 | 0.18 | 0.26 | 0.21 | 0.18 | 0.25 |
| 2012 | 0.17 | 0.13 | 0.21 | 0.17 | 0.13 | 0.20 |
| 2013 | 0.20 | 0.17 | 0.24 | 0.20 | 0.18 | 0.23 |
| 2014 | 0.22 | 0.19 | 0.26 | 0.22 | 0.19 | 0.26 |
| 2015 | 0.22 | 0.19 | 0.26 | 0.22 | 0.18 | 0.25 |
| 2016 | 0.30 | 0.26 | 0.34 | 0.30 | 0.26 | 0.33 |
| 2017 | 0.35 | 0.31 | 0.39 | 0.35 | 0.31 | 0.38 |
| 2018 | 0.33 | 0.29 | 0.37 | 0.32 | 0.29 | 0.35 |
| **Random effects: Variances** | | | | | | |
| Stratum-Level | 0.67 | 0.51 | 0.84 | 0.05 | 0.04 | 0.07 |
| Individual-Level | 3.29 | - | - | 3.29 | - | - |
| **Summary Statistics** | | | | | | |
| VPC | 17% | - | - | 1.4% | - | - |
| PCV | - | - | - | 92% | - | - |
| Reference categories: Male; 21-24; High school or less; year 2000  CI = Confidence Intervals, VPC = Variance Partition Coefficient, PCV = Proportional Change in Variation | | | | | | |

| **Supplementary table 4. Model coefficients and variance (GPD)** | | | | | | |
| --- | --- | --- | --- | --- | --- | --- |
|  | **Null model** | | | **Main effects model** | | |
|  | **est.** | **lower CI** | **upper CI** | **est.** | **lower CI** | **upper CI** |
| **Fixed effects: Regression Coefficients** | | | | | | |
| **Intercept** | 0.57 | 0.43 | 0.66 | 1.62 | 1.51 | 1.76 |
| **Sex/ gender** |  |  |  |  |  |  |
| Female | - | - | - | -0.98 | -1.07 | -0.91 |
| **Age** |  |  |  |  |  |  |
| 25-59 | - | - | - | -0.41 | -0.65 | -0.31 |
| 60+ | - | - | - | -0.66 | -0.78 | -0.55 |
| **Race and ethnicity** |  |  |  |  |  |  |
| Hispanic | - | - | - | -0.46 | -0.61 | -0.31 |
| NH Black | - | - | - | -0.64 | -0.81 | -0.48 |
| NH Asian | - | - | - | -0.18 | -0.35 | 0.01 |
| NH Multiracial | - | - | - | -0.11 | -0.29 | 0.05 |
| NH AI/AN | - | - | - | -0.41 | -0.56 | -0.26 |
| **Education** |  |  |  |  |  |  |
| Some college | - | - | - | 0.04 | -0.07 | 0.14 |
| 4+ years college | - | - | - | 0.18 | 0.08 | 0.26 |
| **Survey year** |  |  |  |  |  |  |
| 2001 | 0.07 | 0.04 | 0.11 | 0.08 | 0.04 | 0.11 |
| 2002 | 0.05 | 0.01 | 0.07 | 0.05 | 0.01 | 0.09 |
| 2003 | 0.06 | 0.02 | 0.09 | 0.06 | 0.03 | 0.10 |
| 2004 | 0.02 | -0.02 | 0.05 | 0.02 | -0.01 | 0.05 |
| 2005 | 0.12 | 0.08 | 0.15 | 0.12 | 0.08 | 0.16 |
| 2006 | 0.10 | 0.06 | 0.13 | 0.10 | 0.07 | 0.13 |
| 2007 | 0.09 | 0.06 | 0.13 | 0.10 | 0.06 | 0.13 |
| 2008 | 0.11 | 0.07 | 0.14 | 0.11 | 0.07 | 0.14 |
| 2009 | 0.14 | 0.11 | 0.17 | 0.14 | 0.10 | 0.17 |
| 2010 | 0.10 | 0.06 | 0.12 | 0.10 | 0.06 | 0.15 |
| 2011 | 0.09 | 0.05 | 0.13 | 0.10 | 0.07 | 0.13 |
| 2012 | 0.14 | 0.10 | 0.17 | 0.15 | 0.11 | 0.18 |
| 2013 | 0.16 | 0.13 | 0.19 | 0.16 | 0.12 | 0.19 |
| 2014 | 0.18 | 0.15 | 0.21 | 0.18 | 0.15 | 0.22 |
| 2015 | 0.13 | 0.10 | 0.16 | 0.13 | 0.10 | 0.17 |
| 2016 | 0.21 | 0.18 | 0.24 | 0.21 | 0.18 | 0.25 |
| 2017 | 0.22 | 0.19 | 0.24 | 0.22 | 0.18 | 0.26 |
| 2018 | 0.19 | 0.16 | 0.22 | 0.19 | 0.15 | 0.23 |
| **Random effects: Variances** | | | | | | |
| Stratum-Level | 0.41 | 0.32 | 0.51 | 0.04 | 0.03 | 0.06 |
| Individual-Level | 2.98 | 2.96 | 2.99 | 2.98 | 2.96 | 3.00 |
| **Summary Statistics** | | | | | | |
| VPC | 12% | - | - | 1.4% | - | - |
| PCV | - | - | - | 88.7% | - | - |
| Reference categories: Male; 21-24; High school or less; year 2000  CI = Confidence Intervals, VPC = Variance Partition Coefficient, PCV = Proportional Change in Variation | | | | | | |


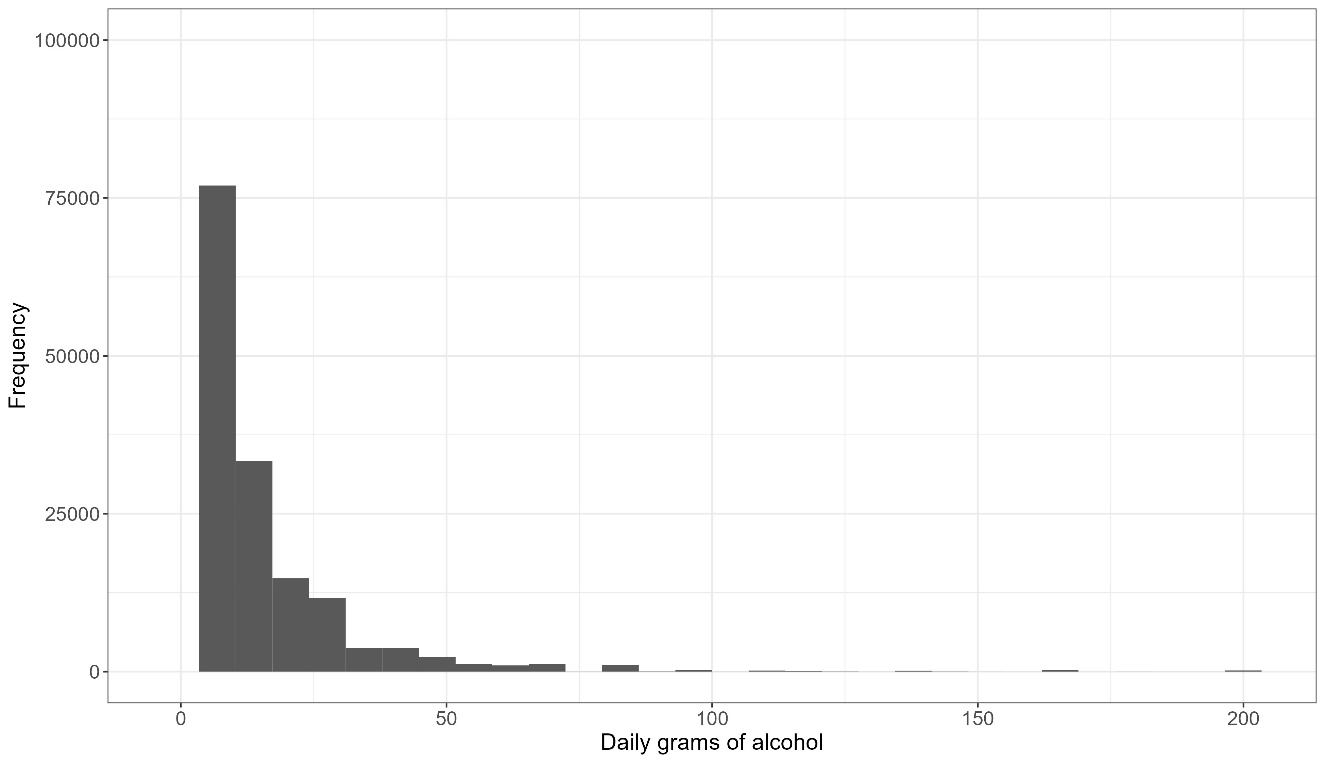


Supplemental figure 1 - Raw distribution of the 'daily grams of alcohol' variable, for current drinkers


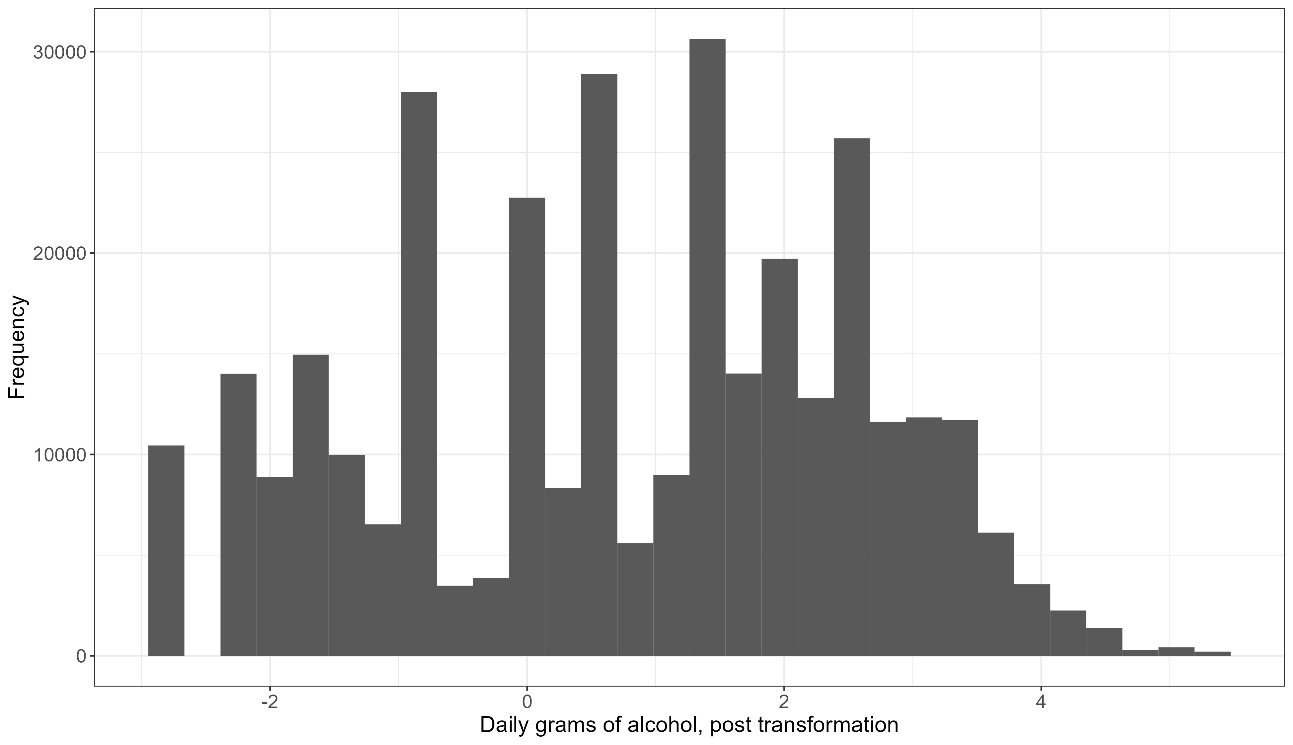


Supplemental figure 2 - Log-transformed distribution of the 'daily grams of alcohol' variable, for current drinkers


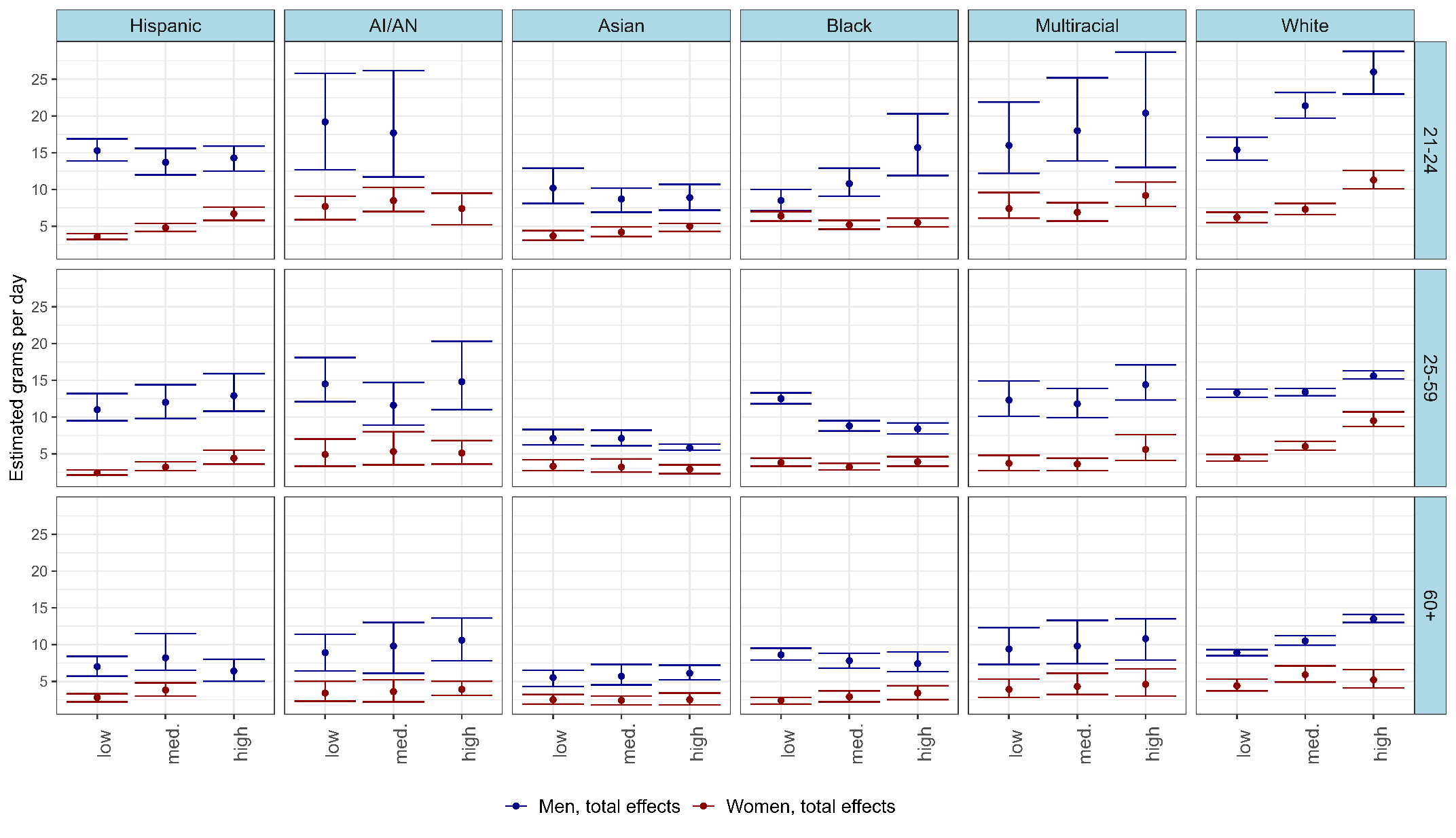


Supplemental Figure 3 - Plot of predicted average alcohol consumption for each intersectional stratum, with error bars indicating 95% credible intervals. Sample restricted to the years 2010-2018.
